# The Diazo-Reaction in Typhoid Fever

**Published:** 1894-01

**Authors:** Julius Friedenwald

**Affiliations:** Baltimore, Md.


### THE DIAZO-REACTION IN TYPHOID FEVER.*

By JULIUS FRIEDENWALD, M. D.,
Baltimore, Md.

The author described the application of the Ehrlich test as follows: Two solutions are prepared:

(*a*) 2 grams sulphanilic acid.
55 c. c. hydrochloric acid.
1000 c. c. distilled water.

(*b*) 0. 5 per cent. solution of nitrite of soda.

In order to perform the reaction 50 parts of *a* and 1 part of *b* are mixed and equal parts of this reagent and of urine placed in a test tube and saturated with ammonia. In those cases in which the reaction is positive, the solution assumes a carmine red color, which on shaking must also be visible in the foam. If the test tube is allowed to stand twenty-four hours a greenish precipitate is found.

Dr. Friedenwald reviewed the literature of the reaction and showed that the objections offered to it depend upon errors in the performance of the test.

(1) Very weak solution of sodium nitrite ($\frac{1}{2}$ per cent.) should be used.

(2) The alcohol test (as proposed by Von Joksch and others) is not to be employed.

(3) A positive reaction is only one in which the red reaction is present in the foam.

Dr. Friedenwald's observations with this reagent includes over three thousand reactions.

Twenty-one cases of typhoid fever were examined; the reaction was absent in but one case. The reaction was obtained as early as the fourth day of the disease and as late as the twenty-sixth. From his observations in typhoid fever he concludes:

(1) That the reaction is very constant in this disease.

(2) That it makes its appearance usually within the first week.

(3) That the reaction gradually disappears between the end of the second and third weeks.

*Abstract of paper read before Clinical Society of Maryland, November 17th, 1893.

Examinations were made in forty-three cases of pulmonary tuberculosis. Of these twenty-nine were severe cases with almost constant reaction, fourteen were light forms which did not show the reaction. Of the twenty-nine severe cases, twelve died while still under observation; eight are still under observation in an unimproved condition.

The presence of the reaction in this disease extending over long periods of time may therefore be regarded as a grave sign.

The reaction was also found to be present in three cases of erysipelas (for several days), one case of bone abscess (tuberculous), one case of liver abscess, one case of suppurative glandular disease of the neck, one case of tubercular hip-joint disease, one case of spinal disease, one case of pneumonia (very grave, died), one case of cancer of the stomach, one case of septicemia.

The reaction was never obtained in healthy individuals nor in many disorders of various kinds, including twenty-one cases of malaria, eleven cases of diabetes, forty-two cases of gastro-intestinal catarrhs. Dr. Friedenwald's observations therefore entirely confirm those of Ehrlich. He concluded with emphasizing Ehrlich's statements:

(1) The diazo-reaction is of great diagnostic value in typhoid fever.

(2) If the cases show a slight or no reaction between the fifth and eighth days, other appearances pointing to typhoid fever, it can be looked upon at once as an exceedingly light form and the prognosis made accordingly.

(3) Gastro-intestinal catarrhs, accompanied by fever, always run their course without a reaction.

(4) Very marked and constant reaction may accompany mild forms of typhoid fever and do not justify a bad prognosis.

(5) Reactions appearing continuously for a long time in phthisis pulmonalis (two months) always indicate a grave prognosis.

[In the discussion which followed this paper Dr. John Hewetson reported the use of Ehrlich's test at the Johns Hopkins Hospital. They had just finished a series of 229 cases treated during the past four years. Of these the test was carefully made in only the last 196, and the reaction obtained in 136 instances. As many patients, however, came to us in the third or fourth week of the disease, it is not fair to say that the reaction was absent in 30 per cent. of our cases, for it may have been present in the earlier stages.

Indeed it is quite common to find it disappear from the urine after the second week. We are not able to give statistics as regards the time of appearance, duration, etc., from the entire number, but from a study of fifty cases it occurred in 77 per cent. in the first week, 67 per cent. in the second, 47 per cent. in the third and in 17 per cent. in the fourth, occurring less frequently after this period, being, however, quite often present during a relapse. As far as I can remember the reaction was in no case obtained before the fourth day. It was frequently found in tuberculosis—34 per cent. of all cases showing the reaction—and in pneumonia, measles and some cases of malarial fever. The reaction seems to fail us just where we need it most, *i. e.*, in differentiating typhoid fever from acute miliary tuberculosis.

It is a sign, however, which we cannot ignore, as is shown in one case which I shall briefly relate. A patient was admitted to the hospital in March of 1892 with symptoms of a severe enterocolitis, the stools very frequently containing blood and mucus. The diarrhea was with difficulty checked. He apparently recovered completely and was discharged in April. Five weeks later he returned with a severe diarrhea—five to twenty-four stools in the twenty-four hours—and symptoms which in every way corresponded to his previous attack. The temperature did not range high, only once or twice touching 102 degrees. His general condition in every way resembled that of his first admission, except that his urine showed a distinct diazo-reaction which persisted until death, which occurred five days after entrance. Autopsy showed typical typhoid lesions, the glands in the small and large intestine being swollen and infiltrated. This, then, would go to support Von Noordin's view, that the cases of gastro-intestinal catarrh with a persistent diazo-reaction in the urine, should be regarded as cases of typhoid fever, but we have not seen it present frequently enough to warrant our giving a decided opinion on the subject.

The degree of value to be attached to the presence of this reaction in the urine for diagnostic purposes is a question. That it is an aid seems undoubted, and we cannot afford to neglect anything which may assist us in this often extremely difficult question.

